# Highly Sensitive Tactile Shear Sensor Using Spatially Digitized Contact Electrodes

**DOI:** 10.3390/s19061300

**Published:** 2019-03-15

**Authors:** Eunsuk Choi, Soonhyung Hwang, Yousang Yoon, Hojun Seo, Jusin Lee, Seongoh Yeom, Gunwoo Ryu, Heewon Yang, Sunjin Kim, Onejae Sul, Seung-Beck Lee

**Affiliations:** 1 Department of Electronic Engineering, Hanyang University, 222 Wangsimni-ro, Seongdong-gu, Seoul 04763, Korea; silver77@hanyang.ac.kr (E.C.); hsh8701@gmail.com (S.H.); ysyoon88@hanyang.ac.kr (Y.Y.); masiks@hanyang.ac.kr (H.S.); jusin19@hanyang.ac.kr (J.L.); yso526@hanyang.ac.kr (S.Y.); rgw00@hanyang.ac.kr (G.R.); heewon0820@hanyang.ac.kr (H.Y.); akangel0307@hanyang.ac.kr (S.K.); 2 Institute of Nano Science and Technology, Hanyang University, 222 Wangsimni-ro, Seongdong-gu, Seoul 04763, Korea; ojsul@hanyang.ac.kr

**Keywords:** shear sensor, high sensitivity, friction detection, surface roughness detection

## Abstract

In this article, we report on a highly sensitive tactile shear sensor that was able to detect minute levels of shear and surface slip. The sensor consists of a suspended elastomer diaphragm with a top ridge structure, a graphene layer underneath, and a bottom substrate with multiple spatially digitized contact electrodes. When shear is applied to the top ridge structure, it creates torque and deflects the elastomer downwards. Then, the graphene electrode makes contact with the bottom spatially digitized electrodes completing a circuit producing output currents depending on the number of electrodes making contact. The tactile shear sensor was able to detect shear forces as small as 6 μN, detect shear direction, and also distinguish surface friction and roughness differences of shearing objects. We also succeeded in detecting the contact slip motion of a single thread demonstrating possible applications in future robotic fingers and remote surgical tools.

## 1. Introduction

When tactile sensors are used for robotic or biomedical applications, they are used to ‘feel’ the tactile environment [1,2]. The resulting signals from tactile shear and slip sensors would act as triggers for certain predetermined actions, such as termination of mechanical movement, initiation of an electrical function, or adjustment of applied mechanical pressure. In these cases, the tactile pressure or shear sensors are acting as level indicators. They are used to determine, at the moment of contact, whether a threshold pressure has been reached, whether the contact pressure has changed to a predetermined level, or a sudden change in pressure at the contacting surface, which is detected as a shear event [3,4]. Therefore, the measurement values returned by the sensor should have a high signal-to-noise ratio and be highly reliable at very low levels of pressure and shear, which is required for controlling sophisticated robotic movements or monitoring the interaction between remote biomedical tools and patients [5,6,7].

Currently, various types of experimental tactile sensors are being reported that have extremely high sensitivity with abilities ranging from tactile shape recognition to insect landing detection [8,9,10,11,12]. For the actual application of these tactile sensors to robotics or biomedical equipment, however, they must be reliable and be able to detect shear and slip, as well as contact pressure, making object manipulation or tactile discrimination possible. There have been sensors developed that detect vertical pressure and lateral shear forces simultaneously, but this simultaneous detection made it difficult to distinguish the signals [11,12,13,14]. To resolve this issue, sensor systems have been developed to distinctively sense shear force using separate dedicated sensors that are integrated into the system. For example, capacitive sensors that detect pressure and shear by measuring the differential capacitance [15,16,17], sensors that use a bump structure on their surface to measure the difference in torsional strain to determine shear forces [18,19], and sensors that use the piezoresistive response difference of a deflected cantilever or beam to determine shear forces [20,21,22,23]. Normally, to enhance the spatial resolution of tactile sensors, one would need to reduce the sensor dimensions for large area integration. This adversely affects most tactile sensors due to an increase in noise and reduced reliability. Capacitance sensor arrays, with their reduced capacitor surface area would be highly susceptible to noise from the slightest changes in the surface environment and from parasitic coupling with adjacent cells [24]. For Si micro-electromechanical systems (MEMS) type piezoresistive sensor arrays, scaling the sensor dimension changes the sensitivity and range of the sensors drastically due to the change in their piezoresistive properties [25]. Thus, a tactile shear sensor that is immune to external noise at reduced dimensions and that does not suffer from active material instability is needed.

In this report, we introduce a highly sensitive tactile shear sensor with spatially digitized contact electrodes (CEs). The tactile shear sensor consists of a suspended elastomer diaphragm with a graphene flexible electrode layer on its bottom surface and spatially digitized electrodes distributed within the open cavity formed by the spacer layer on the substrate. When tactile interaction with the sensor surface leads to lateral shear, the top elastomer deflects and the graphene makes contact with the substrate electrodes. The tactile shear sensor, with a 100 × 100 μm^2^ area, produces variable output currents depending on the number of electrodes making contact with the graphene layer. The sensor was able to detect tactile shear direction and produce surface roughness dependent on the output signals. Also, the contact slip of a gripped single thread was detected, demonstrating the possibility of the tactile shear sensor being applied as sensors in future robotic fingers and remote surgical tools.

## 2. Device Concept and Operating Mechanism

**Figure 1:**
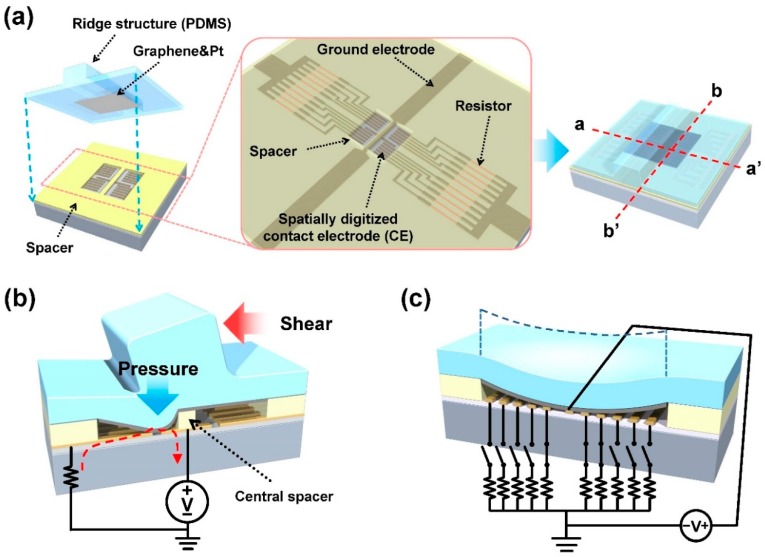
Schematic illustration and operating mechanism of the tactile shear sensor: (**a**) schematic diagram of the sensor; (**b**) 3D cross-sectional image along a-a’ in (**a**), showing the shear sensing mechanism; (**c**) 3D cross-sectional image along b-b’ in (**a**), showing a short-circuit with multiple electrodes making contact with the graphene layer.

Figure 1a shows a schematic diagram of the tactile shear sensor. Its operational concept is similar to the stepped output tactile sensors we have reported previously [26], except that it has a central spacer that supports the top diaphragm directly under a ridge structure on its surface. The sensor consists mainly of two parts, the upper diaphragm with a ridge structure and the bottom substrate. A ground electrode is placed at the center of the pit on the bottom substrate. Multiple spatially digitized CEs are placed at different distances from the ground electrode. The graphene layer was used as a flexible electrode that connected the ground electrode and the CEs under shear application for its mechanical stability under strain. Considering the designed pit dimension with 45 × 100 μm^2^ area and 200 nm thickness, the maximum strain that may be applied to the deflecting diaphragm was calculated to be ~0.9% (=0.4/45). For a flexible electrode on the deflecting diaphragm, graphene with ~25% fracture strain [27] would be more suitable than a metallic thin film such as Au or Pt, which would start to show film cracks under ~1% strain [28,29].

An illustration of the operating concept is shown in Figure 1b,c. Shear forces applied to the ridge structure generate torque around the central spacer, which acts as the axis of rotation, and the torque is converted to a vertical pressure, creating diaphragm deflection. When enough shear is applied, the deflecting graphene on the bottom of the diaphragm completes the circuit between the central ground electrode and the CEs on the substrate, thereby allowing shear to be detected (see Figure 1b). As higher shear is applied, the degree of diaphragm deflection becomes higher, increasing the number of electrodes being shorted, and raising the sensor’s current level output (see Figure 1c). Since the bottom electrode configuration is mirrored about the central spacer, the sensor is able to detect the direction as well as the degree of shear applied. Resistors connected in series to the CEs allow for a higher level of current output difference leading to enhanced shear detection sensitivity.

## 3. Fabrication Process

**Figure 2:**
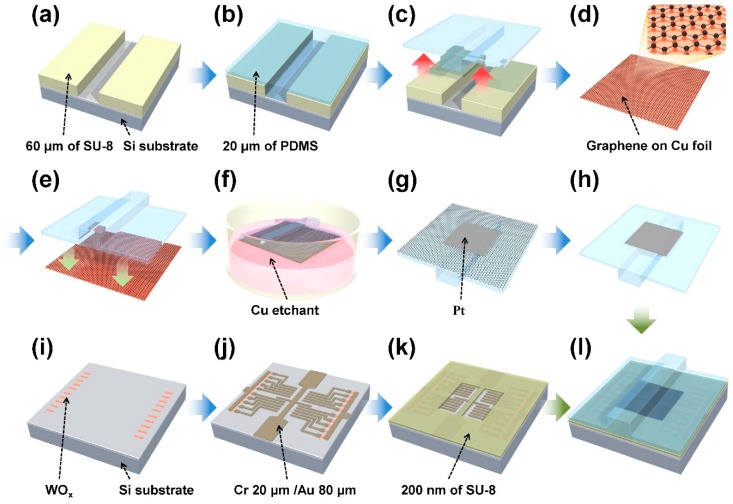
The fabrication process of the tactile shear sensor: (**a**) forming the SU-8 master for the top ridge structure; (**b**) polydimethylsiloxane (PDMS) molding and curing; (**c**) peel-off; (**d**) graphene growth; (**e**) pressing PDMS to graphene; (**f**) Cu foil etching; (**g**) Pt deposition; (**h**) graphene etching; (**i**) resistor formation; (**j**) electrode deposition; (**k**) spacer formation; (**l**) combining the top layer with the bottom substrate.

Figure 2 shows illustrations of the fabrication process. The fabrication process can be divided into two parts, one for the top layer with a ridge structure and the other for the bottom substrate. For the top layer (see Figure 2a–h), we used a molding technique starting with a silicon substrate that had a 60 μm thick SU-8 ridge master pattern defined using optical lithography. Then, a 20 μm thick polydimethylsiloxane (PDMS) was spin-coated on the master and cured. The CVD-grown graphene on Cu foil (Graphene Square Inc., Seoul, Korea) was transferred to the bottom of the ridge-molded PDMS layer. A thin layer (20 nm) of Pt was deposited using a stencil mask and thermal evaporation to reduce the contact resistance and to increase the mechanical stability under repeated contact operation. Then the graphene was etched by O_2_ reactive ion etching, using an evaporated Pt electrode as a hard mask. The bottom substrate fabrication processes (see Figure 2i–k) begin with the defining of the WO_x_ resistors (~1.7 kΩ). The resistors were patterned by electron-beam (e-beam) lithography and 30 nm of W was sputtered in an oxygen atmosphere. The CEs and ground electrode were also patterned using e-beam lithography and 20 nm Cr/80nm Au that was deposited by thermal evaporation. A 200 nm thick spacer was fabricated with SU-8 2002 diluted to 1/4 in SU-8 thinner. Commonly, SU-8 is used as a negative photoresist. However, SU-8 can occasionally be used as a negative e-beam resist due to its cross-linking characteristics induced by the e-beam [30]. Finally, as shown in Figure 2l, the fabricated top layer was aligned and attached to the patterned bottom substrate.

## 4. Fabrication Result and Base Operating Characteristics

**Figure 3:**
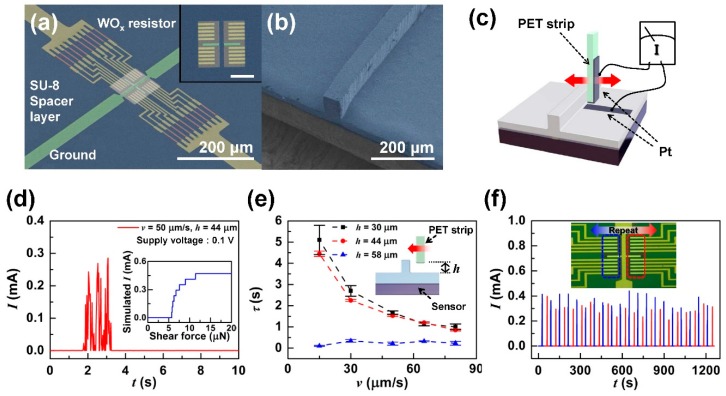
Basic characteristics of the tactile shear sensor: (**a**) false color scanning electron microscope (SEM) image of the fabricated bottom substrate that shows spatially digitized electrodes (yellow), resistors (red), ground electrodes (green) and spacers (blue). Inset image shows the false color SEM image of the spacer pit region taken at low accelerating voltage (tool bar indicates 50 µm); (**b**) PDMS ridge structure with 30 µm width and 60 µm height; (**c**) schematic diagram of the measurement scheme for detecting contact height; (**d**) measurement result of the sensor by polyethylene teraphtalate (PET) strip scanning at 50 µm/s scanning speed *v* and 44 µm contact height *h*. Inset figure shows the simulated output current of the sensor depending on shear magnitude; (**e**) signal duration *τ* of the sensor dependent on *h* and *v*; (**f**) real-time sensor output depending on alternating left (blue) and right (red) shear.

A scanning electron microscope (SEM) image (false color) of the fabricated bottom substrate of the tactile shear sensor is shown in Figure 3a. The spatially digitized CEs were placed symmetrically on either side of the central ground electrode as well as above and below the ground electrode wing. The CEs were arranged with each electrode width and space being 4 μm, with the ground electrode wing placed in the center of the pit. This makes the closest CE–ground spacing 4 μm, with the next closet being 8 μm from the ground electrode. A 100 × 100 μm^2^ pit centered on the ground electrode was patterned in the 200 nm thick SU8 photoresist, with the spacer formed on top of the ground electrode. The PDMS ridge structure on the sensor surface was 60 μm in height and 30 μm in width and is shown in Figure 3b.

To test the sensor’s basic shear detection operation, we used a 125 μm thick polyethylene teraphtalate (PET) strip and scanned it over the sensor ridge structure. To set the scanning height over the sensor surface, a Pt electrode was patterned on the sensor surface and on the bottom of the PET strip (see Figure 3c). The PET strip was lowered until a current was detected and then raised to the measuring height. The contact height and contact speed were controlled by a motorized stage that controlled the PET strip motion. Figure 3d shows the measured sensor current induced by a PET tip scanning at a speed of 50 μm/s over the ridge structure at a height of 44 μm. The tactile shear sensor was designed to produce an output current with magnitude depending on the number of bottom CEs making contact with the ground electrode via the graphene layer. The detected current levels represent the degree of diaphragm deflection induced by the shear force on the ridge structure. Therefore, the observed current fluctuations were caused by the stick–slip interaction between the PET strip scanning over the sensor ridge structure. The detected current magnitudes were between 0~0.28 mA (the supply voltage was 0.1 V). Considering that the resistance of the WO_x_ resistor was ~1.7 kΩ, it was estimated that the number of shorted CEs was five at maximum, which indicates that the contact area of the diaphragm was 44~48 μm. The inset in Figure 3d shows the simulated (COMSOL Multiphysics 5.3a, Comsol Inc., Burlington, MA, USA) result of the relationship between the applied shear force and the output current. It was deduced that the applied shear force was about 7 μN at maximum from the simulation. The simulation showed a stepped output current, since the resistance of the contact was considered to be uniform, whereas in the actual sensor, the varying contact area with varying applied shear force would cause the contact resistance to change, producing shear dependent currents. This result demonstrated that our sensor had the highest shear sensitivity of 1.23 μN^−1^ in the range of 6~11 μN, compared to the shear sensitivities of previously reported sensors: 29.88 N^−1^ [13]; −2.21 N^−1^ [14]; 16.7 N^−1^ [15]. This high shear sensitivity was the result of the small spacing between CEs, which can be sequentially and rapidly contacted by the low shear levels.

To determine how the contacting height on the ridge structure and how the contact speed affects sensor output characteristics, we investigated the sensor’s response under various contact speeds and heights. Figure 3e shows the duration *τ* of the sensor current responses with various contact speeds *v* and PET contact heights *h*. Each scan was repeated five times. For *h* = 30 μm and 44 μm, we observed that the higher the contact speed, the lower the signal duration, which reflects the reduced contact duration with contact speed. When *h* = 58 μm, we observed that *τ* was very low regardless of *v*. This was due to the minimal frictional force between the ridge and the strip, resulting in insufficient torque applied to the ridge. When shear scanning was repeated back and forth, the right and left CEs detected the shear, alternatively demonstrating that the tactile shear sensor was able to detect 6~11 μN shear forces in both directions (Figure 3f). The slightly lower average level of output current for the right scan direction may be from the Pt deposited on the right surface of the PET reducing friction with the PDMS ridge.

## 5. Friction and Roughness Detection

**Figure 4:**
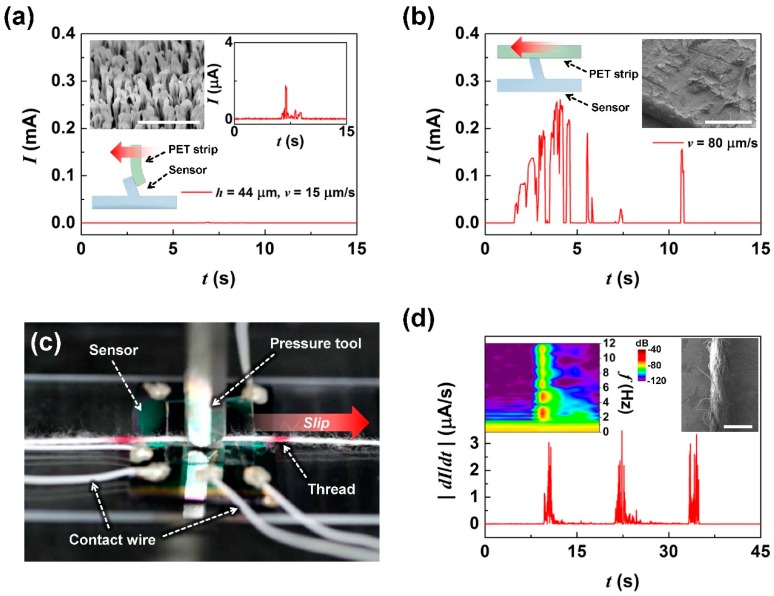
Friction, roughness and slip-sending characteristics of the tactile shear sensor: (**a**) low friction PET with nanobrush scanning result. Inset shows the SEM image of nanobrushes (tool bar indicates 1 μm); (**b**) rough PET surface scanning result. Rough PET surface formed by rubbing with sandpaper. Inset shows an SEM image of the rough PET surface (tool bar indicates 300 μm); (**c**) optical image of the measurement set-up testing the sensor’s slip motion detection capability using a polyester thread; (**d**) absolute value of differential current measured during the slip motion of the thread repeated three times. Left inset shows the short-time Fourier transform (STFT) result at 0~18 s. Right inset shows the SEM images of a polyester thread (tool bar indicates 1 mm).

Based on the shear detection characteristics of the tactile shear sensor, we investigated the effects of the friction and roughness of contact objects on the detected shear levels. Figure 4a shows the sensor output when a low friction PET strip with surface nanobrush structures (see Figure 4a inset SEM image), was scanned on the sensor surface with *v* = 15 μm/s and *h* = 44 μm. The nanobrush structure on PET was fabricated by physical plasma etching with CF_4_ [31,32]. The PET strip with nanobrushes showed a low frictional coefficient due to the highly-reduced real contact area. The friction force *F* for a nanostructure is determined by the relation *F* = *τ* × *A_r_*, where *τ* is the shear strength of the interface, and *A_r_* is the real contact area [33]. The measured frictional coefficient of the PET strip with nanobrushes was about 0.04, in contrast to that of pristine PET, which was about 0.2. The scanning of the pristine PET strip induced 0.26 mA of output current, as shown in Figure 3d, however the scanning of the nanobrushed PET induced less than 2 μA (see Figure 4a inset figure). We can gather that the tactile shear of the nanobrushed PET on the sensor produced minimal diaphragm deflection, enabling only one CE to make contact.

Figure 4b shows the output of the tactile shear sensor when a rough PET strip was horizontally slipped on the sensor surface. The rough PET strip was prepared by rubbing sandpaper on the PET surface at pressure. This produced a rough surface with structures with tens of micron dimensions on their surface, as can be seen in the inset SEM image in Figure 4d. The rough PET strip made contact with the top of the ridge with 100 Pa pressure and was scanned at a speed of 80 μm/s. During shear, the sensor showed irregular output currents changing between 0~0.26 mA. This aperiodic current response was created by the interaction of the irregular hump structure of the rough PET strip with the sensor ridge, which generated aperiodic torque resulting in fluctuating numbers of CE shortings. Thus, we concluded that differences in contact surface friction and roughness were also detectible using the tactile shear sensor.

For applications that require the detection of grip and slip, most systems rely on the time dependent variation in the detected tactile pressure to infer that a slip event has occurred [3,4]. Here, we demonstrate that our sensor is able to detect the pulling of a softly held thread, without the need to detect changes in tactile pressure. We placed a polyester thread on the sensor surface perpendicularly to the ridge structure with 100 Pa vertical pressure (see Figure 4c), mimicking a string being held at minimal force between two fingers. When the polyester thread was pulled with no change in the applied vertical pressure, resulting in slip (at 2 mm/s speed for 2 s period in 10 s of intervals), we measured the time dependent fluctuation in the sensor’s current output, coinciding with the moment of slip, as can be seen in Figure 4d, demonstrating the sensor’s ability to detect slip. For a more detailed analysis, we converted the absolute value of the time-dependent current differential to the signal frequency amplitude using short-time Fourier transform (STFT), as shown in the left inset of Figure 4d. In the 10~12 s of STFT, we observed a primary peak at about 2 Hz and also in its harmonics. Considering the frequency peak *f* determined by the relationship between contact speed *v* and surface period *λ*, *f* = *v*/*λ*, the primary peak can be understood as that caused by ~1 mm periodicity of the surface structure of the thread periodically running over the sensor ridge structure. The measured surface period was confirmed by the observed surface period in the winding of the thread seen from the SEM image of its surface (see the right inset of Figure 4d).

## 6. Discussion

Since the tactile shear sensor’s operational principle does not depend solely on the active sensing element’s material properties, the sensitivity and sensing range can be adjusted by simple modifications to the dimensions of its component parts. The current tactile shear sensor demonstrated a shear force sensitivity of 6 μN, which is only equal to 0.61 mgf (see Figure 3d), but showed a narrow sensing range. Here, the sensor was designed to detect fine slip motion and surface characteristics. As the sensor operation was based on the diaphragm deflection model [34], the sensing range and sensitivity of the tactile shear sensor can be easily tailored by changing the sensor design, e.g. the height and size of the open cavity, the CE interval distance, the number of spatially digitized CEs, and the diaphragm thickness [35]. In principle, it would be difficult for our sensor to detect shear forces that are parallel in direction to the ridge structure. If two shear sensors were integrated perpendicularly to each other, shear from different in-plane directions will be detectable. For vertical pressure detection, we could integrate a stepped output tactile sensor [26] that we have developed previously, which utilizes a digitized CE configuration similar to the shear sensor. Therefore, one pressure sensor integrated with two perpendicular tactile shear sensors will be able to detect tactile forces applied from various directions. Since the sensor has a small active sensing area, it may be possible to integrate it on a laparoscopic grasper for applications in minimally invasive surgery. Also, with further research, applications in prosthetic limbs or in robotic fingers could make highly sensitive artificial tactile shear feedback a real possibility [5,6,7].

## 7. Conclusions

We developed a microfabricated, highly-sensitive, tactile shear sensor that can detect minimal tactile shear. The sensor’s operation relies on the shear-dependent deflection of the elastomer diaphragm making contact between top graphene layer and bottom spatially digitized contact electrodes. The tactile shear sensor is favorable for integration due to its small 100 × 100 μm^2^ active sensing area and showed high shear sensitivity, demonstrating the ability to detect shear force as small as 6 μN. Our tactile shear sensor can discriminate the difference in friction and roughness of contact objects. We also demonstrated the sensor’s ability to detect slip motion. With further developments, the tactile shear sensor may be applicable to future robotic fingers and remote surgical tools.
